# Top 10 International Priorities for Physical Fitness Research and Surveillance Among Children and Adolescents: A Twin-Panel Delphi Study

**DOI:** 10.1007/s40279-022-01752-6

**Published:** 2022-08-24

**Authors:** Justin J. Lang, Kai Zhang, César Agostinis-Sobrinho, Lars Bo Andersen, Laura Basterfield, Daniel Berglind, Dylan O. Blain, Cristina Cadenas-Sanchez, Christine Cameron, Valerie Carson, Rachel C. Colley, Tamás Csányi, Avery D. Faigenbaum, Antonio García-Hermoso, Thayse Natacha Q. F. Gomes, Aidan Gribbon, Ian Janssen, Gregor Jurak, Mónika Kaj, Tetsuhiro Kidokoro, Kirstin N. Lane, Yang Liu, Marie Löf, David R. Lubans, Costan G. Magnussen, Taru Manyanga, Ryan McGrath, Jorge Mota, Tim Olds, Vincent O. Onywera, Francisco B. Ortega, Adewale L. Oyeyemi, Stephanie A. Prince, Robinson Ramírez-Vélez, Karen C. Roberts, Lukáš Rubín, Jennifer Servais, Diego Augusto Santos Silva, Danilo R. Silva, Jordan J. Smith, Yi Song, Gareth Stratton, Brian W. Timmons, Grant R. Tomkinson, Mark S. Tremblay, Stephen H. S. Wong, Brooklyn J. Fraser

**Affiliations:** 1 Centre for Surveillance and Applied Research, Public Health Agency of Canada, 785 Carling Ave, Ottawa, ON K9A 0K9 Canada; 2 School of Epidemiology and Public Health, University of Ottawa, Ottawa, ON Canada; 3 Healthy Active Living and Obesity Research Group, Children’s Hospital of Eastern Ontario Research Institute, Ottawa, ON Canada; 4 School of Human Kinetics, University of Ottawa, Ottawa, ON Canada; 5 Faculty of Health Sciences, Klaipeda University, Klaipeda, Lithuania; 6 Department of Sport, Food and Natural Sciences. Western, Norway University of Applied Science, Bergen, Norway; 7 Population Health Sciences Institute, Newcastle University, Newcastle upon Tyne, UK; 8 Department of Global Public Health and Centre for Epidemiology and Community Medicine (CES), Karolinska Institutet, Stockholm, Sweden; 9 Institute of Management and Health, University of Wales Trinity Saint David, Wales, UK; 10 PROFITH “PROmoting FITness and Health Through Physical Activity” Research Group, Sport and Health University Research Institute (iMUDS), Department of Physical and Sports Education, Faculty of Sport Sciences, University of Granada, Granada, Spain; 11 Canadian Fitness and Lifestyle Research Institute, Ottawa, ON Canada; 12 Faculty of Kinesiology, Sport, and Recreation, University of Alberta, Edmonton, AB Canada; 13 Health Analysis Division, Statistics Canada, Ottawa, ON Canada; 14 Department of Physical Education Theory and Methodology, Hungarian University of Sports Science, Budapest, Hungary; 15 Faculty of Primary and Pre-School Education, ELTE, Eötvös Loránd University, Budapest, Hungary; 16 Kinesiology and Health Science, The College of New Jersey, Ewing, NJ USA; 17 Navarrabiomed, Hospital Universitario de Navarra (HUN), Navarra Institute for Health Research (IdiSNA), Universidad Pública de Navarra (UPNA), Pamplona, Navarra Spain; 18 Department of Physical Education, Federal University of Sergipe, São Cristóvão, SE Brazil; 19 Centre for Population Health Data, Statistics Canada, Ottawa, ON Canada; 20 School of Kinesiology and Health Studies, Queen’s University, Kingston, ON Canada; 21 Department of Public Health Sciences, Queen’s University, Kingston, ON Canada; 22 Faculty of Sport, University of Ljubljana, Ljubljana, Slovenia; 23 Hungarian School Sport Federation, Budapest, Hungary; 24 Research Institute for Health and Sport Science, Nippon Sport Science University, Tokyo, Japan; 25 School of Exercise Science, Physical and Health Education, University of Victoria, Victoria, BC Canada; 26 School of Physical Education and Sport Training, Shanghai University of Sport, Shanghai, China; 27 Shanghai Research Center for Physical Fitness and Health of Children and Adolescents, Shanghai University of Sport, Shanghai, China; 28 Department of Biosciences and Nutrition, Karolinska Institutet, Stockholm, Sweden; 29 Department of Health, Medicine and Caring Sciences, Linköping University, Linköping, Sweden; 30 Centre for Active Living and Learning, College of Human and Social Futures, The University of Newcastle, Callaghan, NSW Australia; 31 Baker Heart and Diabetes Institute, Melbourne, VIC Australia; 32 Research Centre of Applied and Preventive Cardiovascular Medicine, University of Turku, Turku, Finland; 33 Centre for Population Health Research, Turku University Hospital, University of Turku, Turku, Finland; 34 Menzies Institute for Medical Research, University of Tasmania, Hobart, TAS Australia; 35 Division of Medical Sciences, University of Northern British Columbia, Prince George, BC Canada; 36 Department of Health, Nutrition, and Exercise Sciences, North Dakota State University, Fargo, ND USA; 37 Fargo VA Healthcare System, Fargo, ND USA; 38 Laboratory for Integrative and Translational Research in Population Health (ITR), Research Center in Physical Activity, Health and Leisure (CIAFEL), Faculty of Sports, University of Porto (FADEUP), Porto, Portugal; 39 Alliance for Research in Exercise, Nutrition and Activity (ARENA), University of South Australia, Adelaide, SA Australia; 40 Murdoch Children’s Research Institute, University of Melbourne, Parkville, Melbourne, VIC Australia; 41 Department of Physical Education, Exercise and Sports Science, Kenyatta University, Nairobi, Kenya; 42 PROFITH “PROmoting FITness and Health Through Physical Activity” Research Group, Sport and Health University Research Institute (iMUDS), Department of Physical and Sports Education, Faculty of Sport Sciences, University of Granada, Granada, Spain; 43 Faculty of Sport and Health Sciences, University of Jyväskylä, Jyväskylä, Finland; 44 Department of Physiotherapy, University of Maiduguri, Maiduguri, Nigeria; 45 Facultad de Ciencias de la Educación, Unidad Central del Valle del Cauca (UCEVA), Túlua, Colombia; 46 CIBER of Frailty and Healthy Aging (CIBERFES), Instituto de Salud Carlos III, Madrid, Spain; 47 Department of Physical Education and Sport, Technical University of Liberec, Liberec, Czech Republic; 48 Institute of Active Lifestyle, Palacký University Olomouc, Olomouc, Czech Republic; 49 Sports Center, Federal University of Santa Catarina, Florianópolis, SC Brazil; 50 Institute of Child and Adolescent Health, School of Public Health, National Health Commission Key Laboratory of Reproductive Health, Peking University, Beijing, China; 51 Applied Sport Technology Exercise and Medicine Research Centre, Faculty Science and Engineering, Swansea University, Wales, UK; 52 Child Health and Exercise Medicine Program, McMaster University, Hamilton, ON Canada; 53 Department of Education, Health and Behavior Studies, University of North Dakota, Grand Forks, ND USA; 54 Department of Pediatrics, University of Ottawa, Ottawa, ON Canada; 55 Department of Health Sciences, Carleton University, Ottawa, ON Canada; 56 Department of Sports Science and Physical Education, The Chinese University of Hong Kong, Shatin, Hong Kong; 57 Faculty of Health Science, Universidad Autónoma de Chile, Santiago, Chile

## Abstract

**Background:**

The measurement of physical fitness has a history that dates back nearly 200 years. Recently, there has been an increase in international research and surveillance on physical fitness creating a need for setting international priorities that could help guide future efforts.

**Objective:**

This study aimed to produce a list of the top 10 international priorities for research and surveillance on physical fitness among children and adolescents.

**Methods:**

Using a twin-panel Delphi method, two independent panels consisting of 46 international experts were identified (panel 1 = 28, panel 2 = 18). The panel participants were asked to list up to five priorities for research or surveillance (round 1), and then rated the items from their own panel on a 5-point Likert scale of importance (round 2). In round 3, experts were asked to rate the priorities identified by the other panel.

**Results:**

There was strong between-panel agreement (panel 1: *r*_s_ = 0.76, *p* < 0.01; panel 2: *r*_s_ = 0.77, *p* < 0.01) in the priorities identified. The list of the final top 10 priorities included (i) “conduct longitudinal studies to assess changes in fitness and associations with health”. This was followed by (ii) “use fitness surveillance to inform decision making”, and (iii) “implement regular and consistent international/national fitness surveys using common measures”.

**Conclusions:**

The priorities identified in this study provide guidance for future international collaborations and research efforts on the physical fitness of children and adolescents over the next decade and beyond.

**Supplementary Information:**

The online version contains supplementary material available at 10.1007/s40279-022-01752-6.

## Key Points

**Table:** 

Physical fitness among children and adolescents is an important marker of current and future health. Considering declines in some aspects of physical fitness among children and adolescents, there is a need to set international priorities for research and surveillance to help guide future efforts.
Using a twin-panel Delphi method, two panels identified 36 (panel 1) and 25 (panel 2) research or surveillance priorities. The between-panel agreement was strong, leading to a combined list of the top 10 overall priorities.
The top three priorities identified were the need to (1) “conduct longitudinal studies to assess changes in fitness and associations with health”, (2) “use fitness surveillance to inform decision making”, and (3) “implement regular and consistent international/national fitness surveys using common measures”.

## Introduction

Physical fitness consists of multiple components such as cardiorespiratory fitness (CRF), musculoskeletal fitness (MSF; i.e., muscular strength, power, endurance, and flexibility), agility, speed, balance, coordination, and body composition, which collectively reflect an individual’s ability to perform physical activity [1]. Measurement of physical fitness has a long history that dates back more than 200 years to Adolphe Quételet, a pioneer in anthropometry [2, 3]. In 1835, Quételet began measuring the handgrip strength of Belgian boys and girls [4, 5]. From the early 1900s, fitness testing of children and adolescents expanded beyond anthropometry and isometric muscle strength to include exercise capacity and motor performance (e.g., sprinting, jumping) [6, 7]. During the two World Wars (1914–1918 and 1939–1945) there was an international focus on measuring and improving performance-related fitness (i.e., having the skills and physical abilities to engage in a competitive environment) for military preparedness [6]. However, in the 1970s, because of research demonstrating that low physical fitness was significantly associated with poor health outcomes among adults [8, 9], physical fitness testing started to shift from a performance-related to a health-related focus [6]. The evidence supporting health-related fitness (i.e., the fitness components significantly linked with current and future health [6]) among children and adolescents arrived later, with research beginning to appear in the early 1990s for CRF [10, 11] and the early 2000s for MSF [12, 13].

Findings from cross-sectional studies suggest that high CRF and MSF among children and adolescents is associated with a range of health benefits, such as better cardiovascular health, skeletal health, motor competence, cognitive ability, mental health, and self-esteem [11, 12, 14–16]. In addition, CRF levels are a stronger predictor of cardiovascular disease risk factors among youth than objectively measured physical activity levels [17]. Longitudinal epidemiological studies have shown that physical fitness levels persist (i.e., track) across the life course [18–21], and that high CRF and MSF in childhood, adolescence, or early adulthood is prospectively associated with a healthier cardiovascular profile [13, 22–24], reduced disability [25, 26], and a decreased risk of premature mortality [27, 28] in adulthood. An individual’s physical fitness level, especially their CRF, provides a reasonable objective indication of their moderate to vigorous intensity physical activity levels in recent months, as it summarizes the physiological response to their physical activity profile [29]. In addition, physical fitness testing is feasible, cost effective, and suitable for population surveillance [30, 31]. For these reasons, there has been a strong international call to universally measure physical fitness among children and adolescents for global health surveillance, monitoring, and clinical screening [6, 14, 32, 33].

Anthropometric measures (i.e., body mass index, waist circumference) have long been an important indicator of health in research, surveillance, and clinical practice [34]. The same cannot be said for other components of physical fitness (e.g., CRF, MSF) despite mounting evidence of their importance [31]. In light of declining international levels of some aspects of fitness (e.g., CRF, leg power, abdominal/core endurance) among children and adolescents [35–37], there is a need to refocus international efforts to identify the priorities that can help address major literature gaps and guide future physical fitness research and health surveillance. The Delphi method is described as a systematic approach to gather expert opinions and arrive at consensus [38]. This Delphi approach has been previously used to identify priorities in physical activity and sedentary behavior research [39]. Thus, the objective of this research was to conduct a twin-panel Delphi study to determine an international list of the top 10 priorities for physical fitness research and surveillance among children and adolescents over the next decade.

## Methods

### Overview

This study implemented a twin-panel Delphi procedure, which allowed two independent groups (the Delphi panels) of experts to address our research objective based on their subjective opinions [38]. Over the course of several rounds, the Delphi procedure allowed the two expert panels to systematically refine their responses to arrive at a final list of priorities [40]. The twin-panel approach is an improvement from a traditional single-panel Delphi because it allows expert panels to cross-validate the ranked priorities identified by each panel.

### Participant Sampling Strategy

#### Panel 1

Sampling for panel 1 took place as part of a large international fitness meeting hosted by the Public Health Agency of Canada on August 19, 2021. The meeting aimed to discuss and explore potential directions to address international priority areas in fitness research and health surveillance. See the electronic supplementary material (ESM) for a brief outline of the meeting agenda. Meeting delegates (i.e., experts) were selected based on the lead organizers’ (JJL, BJF) knowledge of individuals who were actively engaged in fitness research and surveillance. The final group of attendees included 45 participants: 17 were Canadian fitness experts who worked in policy, programs, or surveillance; 12 were fitness experts from Canadian universities; and 16 were international experts from outside Canada. Academic experts were identified if they had published a peer-reviewed research article that assessed or interpreted youth fitness within the last 5 years. PhD students were considered if their dissertation was directly related to fitness assessment or surveillance. The majority of the meeting participants were invited to participate in the Delphi study, with the final response rate being 62% (28/45).

#### Panel 2

To identify research experts to include as part of panel 2, a SciVal list of the top 100 authors worldwide based on the topic cluster “Cardiorespiratory Fitness; Skinfold Thickness; School Children” (Topic T.7814) was used on August 4, 2021. These experts were then ranked by scholarly output (i.e., the total count of research outputs) to identify the most productive researchers in this SciVal research category. From this list, 10 researchers were excluded because they participated in panel 1. The remaining 57 researchers who had been a first or senior (i.e., last/corresponding) author on a relevant publication and had an h-index of ≥ 5 were invited, with 32% (18/57) agreeing to participate.

### Survey Procedure

The Delphi included three rounds of data collection and analysis. All surveys were created and administered in Google Forms (Mountain View, CA, USA). For each round, participants were provided with a direct web link to the survey via emails. All participants were allowed 3 weeks to complete each round, with a reminder email sent after 2 weeks. All three rounds were completed between August and November 2021. Participants were not required to complete all three rounds to retain their responses. Google Sheets (Mountain View, CA, USA) was used to organize responses and to conduct data analyses. Each panel conducted the Delphi independently, following the same methods. Participants were not made aware of the other panel (i.e., blinded) until round 3. Those who completed all three rounds of the Delphi study were invited to contribute to this research article as a co-author.

#### Round 1

All participants were provided with a cover letter and asked to answer the following question: “In your opinion, what is the most important priority area for physical fitness research and surveillance among children and adolescents that should be addressed over the next 10 years?” Participants were asked to describe the priority in one or two sentences. They were then asked to provide supporting details, such as examples or supporting literature, for the identified priority area. Participants were provided the opportunity to identify five priority areas. One researcher (JJL) reviewed all priorities submitted by the participants. Similar priorities were combined into a single overarching priority theme. A second researcher (BJF) reviewed the priority themes for accuracy. Discussions took place between the two researchers (JJL, BJF) to resolve any disagreement, with a third researcher (GRT) consulted for any unresolved disagreement.

#### Round 2

During round 2, participants were provided with a cover letter and asked to review the list of overarching priority themes identified by their respective panel during round 1. Participants were notified that their responses were merged with similar priority areas to create overarching priority themes that may not directly reflect their original wording. Participants were asked to rate the level of importance over the next 10 years for each priority theme using a 5-point Likert scale (0 = don’t know, 1 = somewhat important, 2 = moderately important, 3 = important, 4 = very important, 5 = extremely important). Mean scores were calculated and ranked in descending order from highest to lowest. The standard deviation was used as a tiebreaker with lower standard deviations being ranked higher. Participants who responded as ‘don’t know’ were coded as a missing value that did not contribute to the denominator in calculating mean scores.

#### Round 3

In round 3, participants were provided with a cover letter and asked to rate the level of importance of the priorities identified by the other panel using the same 5-point Likert scale from round 2. For instance, panel 1 rated the 25 priorities identified by panel 2, and panel 2 rated the 36 priorities identified by panel 1. Like round 2, mean scores were calculated to rank priorities, and standard deviations were used as a tiebreaker.

### Statistical Analysis

Spearman’s rank correlation coefficients were used to assess the level of between-panel agreement in the ranked priorities. Using responses from round 3, one correlation coefficient was calculated for the agreement for panel 1’s ranked priorities, and a second correlation coefficient was calculated for the agreement on panel 2’s ranked priorities. Correlations of 0.1, 0.3, and 0.5 were used as thresholds for weak, moderate, and strong agreement, respectively [41]. To identify the top 10 priorities, an a priori decision was made to combine the ranked lists for panels 1 and 2 using the overall or mean (if the priority was included in both panel lists) Likert scale response from round 2.

## Results

### Participant Demographics

**Fig. 1:**
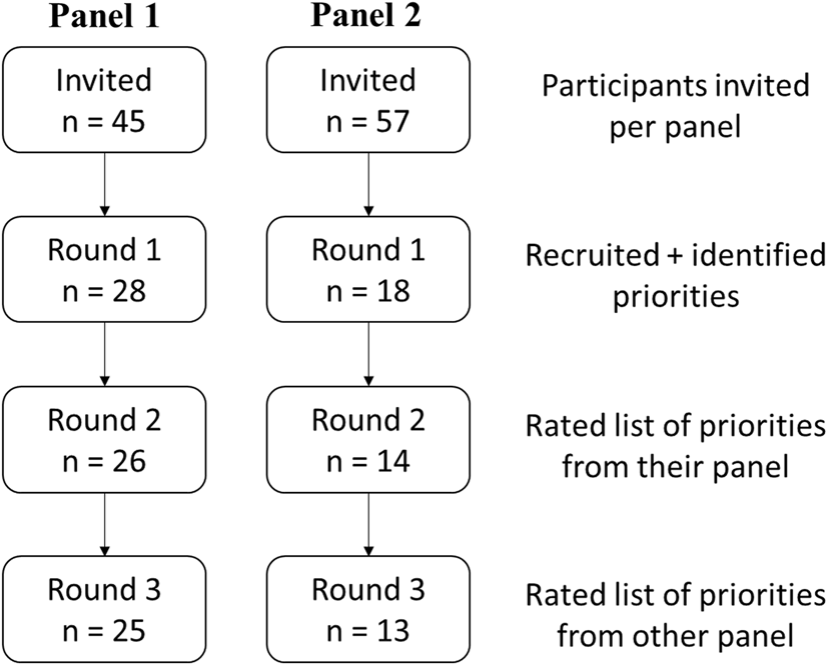
Flow chart depicting the participant retention across all three rounds of the twin-panel Delphi study

Table 1 describes the participant characteristics. Panel 1 included participants from all career stages (0–5 years, 6–10 years, 11–20 years, and 21+ years). The panel 1 participants resided in six continents across all country income levels, with the majority from North America. Panel 2 was smaller and did not include students, or participants living in Africa, or low-income countries. The study retention was strong with 89% (25/28) and 72% (13/18) of panel 1 and 2 participants completing all three rounds of the study, respectively (Fig. 1).

**Table 1:** Descriptive statistics for Delphi study panels during Round 1

	Panel 1 (*n* = 28)	Panel 2 (*n* = 18)
Mean age years (SD)	43.4 (10.6)	47.8 (13.0)
Gender (% female)	8 (28.6%)	4 (22.2%)
**Occupation in 2021**		
Scientist/researcher (e.g., professor, post-doctoral fellow)	23 (82.1%)	17 (94.4%)
Research assistant/research manager	1 (3.6%)	1 (5.6%)
Student (e.g., PhD student)	1 (3.6%)	0 (0%)
Other	3 (10.7%)	0 (0%)
**Career stage (years of experience post-graduation)**		
Current student	1 (3.6%)	0 (0%)
0–5 years	3 (10.7%)	3 (16.7%)
6–10 years	8 (28.6%)	3 (16.7%)
11–20 years	12 (42.9%)	5 (27.8%)
20+ years	4 (14.3%)	7 (38.9%)
**Primary country of occupation**		
North America	14 (50%)	1 (5.6%)
South America	1 (3.6%)	4 (22.2%)
Europe	6 (21.4%)	9 (50.0%)
Africa	2 (7.1%)	0 (0%)
Asia	4 (14.3%)	1 (5.6%)
Oceania	1 (3.6%)	3 (16.7%)
**Primary country GDP (self-reported)**		
High-income	20 (71.4%)	13 (72.2%)
Middle-income	6 (21.4%)	5 (27.8%)
Low-income	2 (7.1%)	0 (0%)

*GDP* gross domestic product, *n* sample size, *SD* standard deviation

### Delphi Results

During round 1, panel 1 submitted 104 unique responses that were qualitatively reduced into 36 unique priority themes (Table 2). Panel 2 submitted 71 responses that were reduced into 25 priority themes (Table 3). Eight priorities overlapped between the panels. An overview of the unique responses by priority theme is provided in the ESM.

**Table 2:** Priority themes identified by Panel 1

Panel 1 ranking	Priority areas	Panel 1 rating, mean (SD)^a^	Panel 2 ranking
1	*Conduct longitudinal studies to assess changes in fitness and associations with health*	4.46 (0.81)	7
2	*Use fitness surveillance to inform decision making*	4.35 (0.69)	4
3	Investigate interventions to improve fitness	4.12 (1.03)	9
4	Assess the reliability and validity of fitness measures	4.12 (1.07)	16
5	Develop a common/universal international field-based fitness test battery	4.12 (1.07)	3
6	Investigate and reduce inequalities in fitness	4.08 (0.76)	14
7	*Implement regular and consistent international/national fitness surveys using common measures*	4.08 (1.13)	1
8	Develop an international fitness data repository	4.04 (1.11)	10
9	*Develop universal health-related fitness cut-points*	4.04 (1.11)	15
10	Increase fitness data in low- and middle-income countries	3.96 (0.93)	18
11	*Better understand how components of fitness impact health*	3.92 (1.02)	2
12	Identify the dose–response relationship between fitness and health	3.92 (1.09)	6
13	Use fitness as a clinical vital sign to monitor for health screening in clinical settings	3.88 (1.39)	13
14	*Develop international/national normative-referenced centile values for fitness*	3.85 (1.08)	8
15	Untangle the health benefits of fitness vs physical activity	3.77 (0.76)	22
16	*Implement school-based fitness monitoring*	3.77 (1.24)	5
17	Use fitness as a primary outcome in research studies that intervene with physical activity	3.69 (1.01)	24
18	Shift from a focus on obesity to a focus on fitness for health	3.69 (1.12)	26
19	*Study the link between fitness and mental/cognitive health*	3.69 (1.26)	11
20	Identify determinants and correlates to help improve fitness among children and youth	3.68 (0.99)	19
21	Measure fitness to help understand physical activity levels in a population	3.68 (1.11)	31
22	Overcome the stigma of fitness testing (i.e., fear of injury)	3.65 (1.26)	33
23	Implement an international fitness survey for those with disabilities	3.64 (0.95)	21
24	Identify the main construct measures of fitness among children and youth	3.60 (1.12)	25
25	Improve international comparison of fitness trends	3.58 (1.10)	12
26	Investigate the associations between motor fitness and health	3.38 (1.17)	30
27	Investigate fitness as a mediator of obesity risk	3.16 (0.94)	35
28	Assess trends in fitness while controlling for adiposity	3.16 (1.25)	20
29	Determine the frequency that fitness should be measured in a population	3.08 (1.26)	32
30	Assess the effect of COVID-19 restrictions on fitness levels	3.08 (1.44)	29
31	Promote the benefits of resistance type training	3.00 (1.22)	17
32	Investigate international trends in obesity	2.96 (1.34)	28
33	Develop field tests that are independent of body size	2.88 (1.36)	27
34	Investigate the role of genetics and the environment on fitness	2.77 (1.24)	23
35	Identify backup fitness measures to use as a proxy when primary measures can't be used	2.50 (1.14)	34
36	Use fitness testing for sport talent identification	1.96 (1.12)	36

*Italicized* priority areas were common between both panels

Priorities are ordered from the most important to least important by the Panel 1 mean ratings from Round 2. The Panel 2 rankings were obtained from Round 3 responses

^a^Data are presented as the mean from a 5-point Likert scale

*SD* standard deviation

**Table 3:** Priority themes identified by Panel 2

Panel 2 ranking	Priority areas	Panel 2 rating, mean (SD)^a^	Panel 1 ranking
1	*Conduct longitudinal studies to assess changes in fitness and associations with health*	4.43 (0.85)	2
2	*Implement regular and consistent international/national fitness surveys using common measures*	4.36 (0.84)	4
3	*Develop universal health-related fitness cut-points*	4.29 (0.73)	5
4	*Study the link between fitness and mental/cognitive health*	4.21 (0.58)	13
5	*Develop international/national normative-referenced centile values for fitness*	4.21 (0.58)	3
6	Implement scalable school-based interventions to improve and promote fitness	4.21 (0.89)	7
7	*Use fitness surveillance to inform decision making*	4.14 (0.86)	1
8	Focus on shifting trends in fitness levels among children	4.00 (0.78)	14
9	Investigate cost effectiveness of interventions aimed at increasing fitness	4.00 (0.88)	19
10	Investigate the causal associations between fitness for health and well-being	4.00 (0.96)	6
11	Improve muscular strength promotion among youth	3.93 (0.92)	20
12	*Implement school-based fitness monitoring*	3.93 (1.14)	9
13	Investigate effective interventions to improve fitness among unfit youth	3.86 (0.86)	12
14	Increase fitness data in low- and middle-income countries and rural areas	3.86 (1.29)	16
15	Implement physical literacy interventions in schools with a focus on fitness	3.79 (1.12)	17
16	*Better understand how components of fitness impact health*	3.79 (1.25)	10
17	Engage stakeholders, funding bodies, NGOs, etc. to understand the importance of fitness	3.71 (1.20)	8
18	Tracking of fitness from childhood to late adolescence	3.36 (1.45)	11
19	Assess physical fitness by socioeconomic status and parental education	3.21 (1.27)	23
20	Establishing consensus on how best to account for body size/shape when measuring fitness	3.14 (1.17)	15
21	Determine if body composition or physical fitness is a better predictor of health outcomes	3.14 (1.29)	18
22	Investigate the parental influence on childhood fitness levels	2.93 (1.21)	22
23	Investigate the genetic determinants of physical fitness	2.86 (1.23)	25
24	Investigate the relationship between sport participation and physical fitness	2.79 (1.12)	21
25	Investigate the link between fitness and nutrition	2.71 (1.44)	24

*Italicized* priority areas were common between both panels

Priorities are ordered from the most important to least important by the panel 2 mean ratings from round 2. The panel 1 rankings were obtained from round 3 responses

^a^Data are presented as the mean from a 5-point Likert scale

*SD* standard deviation

In round 2, participants were asked to rate the level of importance for each priority identified by their respective panel. The mean Likert-scale scores ranged from 1.96 to 4.46 and 2.71 to 4.43 for panels 1 and 2, respectively. Of the eight overlapping priorities, four emerged in the top 10 priorities for panel 1 and six emerged in the top 10 priorities for panel 2. For panel 2, the top five priorities were also identified by panel 1. Both panels identified “conduct longitudinal studies to assess changes in fitness and associations with health” as the number one priority. “Use fitness surveillance to inform decision making”, “implement regular and consistent international/national fitness surveys using common measures”, and “develop universal health-related fitness cut-points” were common priorities that were ranked in the top 10 for both panels.

**Fig. 2:**
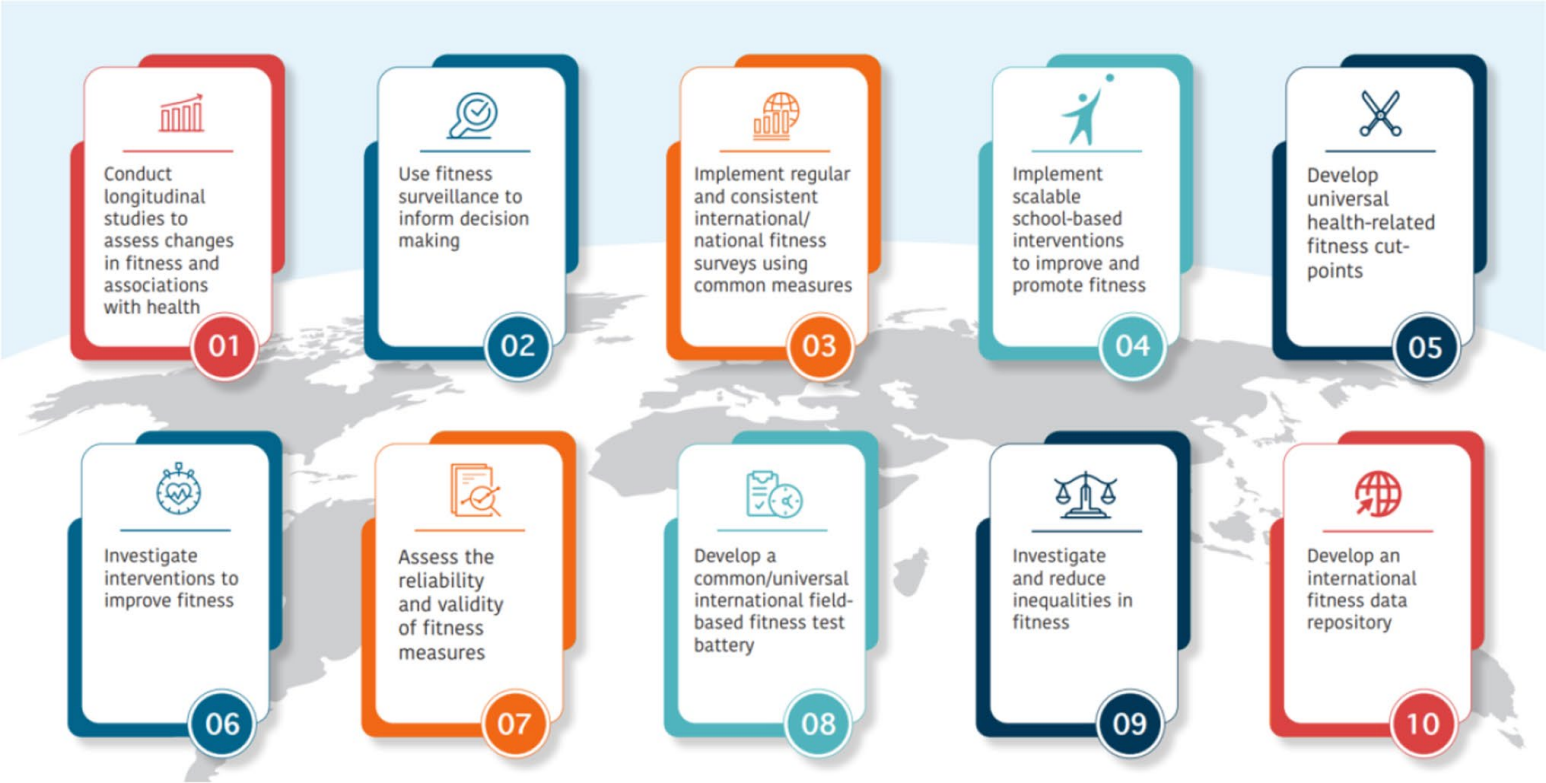
Top 10 international priorities for physical fitness research and surveillance among children and adolescents identified by international experts in fitness

During the final round, expert participants were asked to rate the level of importance for each of the other panel’s priorities. The between-panel agreement was strong for both panel 1 (*r*_s_ = 0.76, *p* < 0.01) and panel 2 (*r*_s_ = 0. 77, *p* < 0.01) using responses from round 3. Given the strong agreement between panels, the priorities identified by both panels were combined to identify the top 10 overall priorities (Table 4, Fig. 2).

**Table 4:** The top 10 priority areas identified by both panels

Ranking	Priority areas	Mean rating^a^	Panel
1	Conduct longitudinal studies to assess changes in fitness and associations with health	4.45	Both
2	Use fitness surveillance to inform decision making	4.25	Both
3	Implement regular and consistent international/national fitness surveys using common measures	4.22	Both
4	Implement scalable school-based interventions to improve and promote fitness	4.21	Panel 2 only
5	Develop universal health-related fitness cut-points	4.17	Both
6	Investigate interventions to improve fitness	4.12	Panel 1 only
7	Assess the reliability and validity of fitness measures	4.12	Panel 1 only
8	Develop a common/universal international field-based fitness test battery	4.12	Panel 1 only
9	Investigate and reduce inequalities in fitness	4.08	Panel 1 only
10	Develop an international fitness data repository	4.04	Panel 1 only

The calculated mean rating (i.e., panel 1 mean + panel 2 mean/2) from priorities that overlapped between panels, or single panel mean rating were used to rank the top 10 priorities from the most important to least important

^a^Data are presented as the mean from a 5-point Likert scale

## Discussion

To our knowledge, this is the first study to have used a twin-panel Delphi method to identify a list of international priority areas for physical fitness research and surveillance among children and adolescents. The top 10 priorities reflect diverse fields of study, from epidemiology to social science, and notably, to achieve many of the priorities, international collaboration is required. Below, we summarize topical evidence related to these ten research priorities.

### Priority 1: Conduct Longitudinal Studies to Assess Changes in Fitness and Associations with Health

In recent decades, several longitudinal studies have established that physical fitness in adolescence is a significant inverse and independent predictor of disease outcomes, including premature mortality in adulthood [13, 23–28]. Some studies on adolescents investigated changes in fitness levels (i.e., CRF and MSF) and associations with health outcomes using follow-up periods of several years [42–44], whilst others identified that improvements in MSF from childhood to adolescence were associated with reduced adiposity [13, 45]. There is a need for future studies to link fitness (both CRF and MSF) measured in young childhood (of both sexes) with clinical outcomes in adulthood in nationally representative cohorts to establish longitudinal links with key health outcomes [13, 22, 24, 27]. Such studies could provide valuable insights into physical fitness and the associated risk of developing and dying from a chronic disease (i.e., relative risk), that could be used to calculate the population attributable fraction. There is also a need to better understand the link between childhood fitness and future mental disorders, given the increasing burden of mental health problems in some countries [46], especially in the context of the COVID-19 pandemic [47]. Furthermore, cohorts with multiple follow-ups allow for an assessment of changes and trajectories in fitness over time which can be used to calculate the meaningful clinically important difference (i.e., what is the minimum improvement in fitness required for meaningful changes in physical health status?). An example is the Aerobic Centre Longitudinal Study cohort for which statistically significant reductions in all-cause and cardiovascular disease mortality were found among men who maintained or improved their physical fitness over a 5-year period [48].

### Priority 2: Use Fitness Surveillance to Inform Decision Making

Public health surveillance is essential to guide health promotion efforts. Many countries collect and report regularly on body composition and self-reported physical activity through national health surveillance systems [49]. However, surveillance systems can be expanded to report on other important measures of physical fitness such as CRF and MSF [32, 50]. Some countries, including Slovenia, Hungary, and Japan, have implemented routine national fitness surveillance [31, 51]. While others, such as Australia, have recently scaled back ongoing national fitness surveillance efforts [52]. National fitness surveillance efforts in Slovenia identified a 13% decline in the fitness levels of youth aged 6–15 years following 2 months of COVID-19-related lockdowns [53]. Other countries used national fitness surveillance to identify regions/groups with low fitness levels and in need of intervention [54]. The approach to incorporate national fitness surveillance efforts have also been used to track the effectiveness of national policy efforts aimed at increasing the physical activity levels of children and youth in the school context [55]. Countries could further benefit from leveraging the measurement of physical fitness (CRF and MSF) to inform and track the effectiveness of policy and programming to improve the health of children and adolescents.

### Priority 3: Implement Regular and Consistent International/National Fitness Surveys Using Common Measures

The 2018 Global Matrix 3.0 of Physical Activity Report Cards for Children and Youth, for the first time, included physical fitness as an indicator [56]. Unfortunately, over half (55%) of the included countries were unable to report a grade for physical fitness due to a lack of available data [56]. This suggests that most countries do not implement regular fitness surveys/testing among children and adolescents. Of the countries that do implement regular fitness surveys, the measurement protocols varied substantially both within and between countries. For instance, CRF is measured nationally using a submaximal step test in Canada, a treadmill test in the USA, and a variety of field-based tests (e.g., the 20-m shuttle run test, distance runs, timed runs) in Japan, Estonia, and Hungary [31, 35]. There is more international consistency with the measurement of MSF (especially for muscular strength, which is commonly assessed as isometric maximal handgrip strength), but still, major international differences in protocols and reporting exist [57]. Implementing regular and consistent international and national fitness surveys, similar to efforts conducted for physical activity [49, 58], would help better describe the global health status of children and adolescents.

### Priority 4: Implement Scalable School-Based Interventions to Improve and Promote Fitness

Many countries have recently observed declines in measures of physical fitness among children and adolescents [35, 36, 59], likely resulting in meaningful reductions in population health. There is a need to promote fitness among children and adolescents using safe, equitable, and inclusive approaches [60]. Although it is not always the case, most youth spend a substantial part of their day in the school environment. As a result, schools provide a unique opportunity to implement interventions aimed at improving fitness (e.g., via increased quality and quantity of physical activity throughout the day [61]). Several systematic reviews have found positive improvements in the physical fitness levels (i.e., MSF and CRF) of children and adolescents associated with school-based interventions [62–65]. More recently, school-based interventions using high-intensity interval training have demonstrated promising improvements for youth CRF and other important health markers [66]. However, gaps and limitations persist. For example, future interventions need to better assess the sustained impact of interventions by including longer follow-up times [63], and their potential scalability while incorporating implementation science frameworks [67]. Future interventions aimed at increasing physical activity in the school environment could use objective measures of physical fitness as the primary study outcome [68]. Lastly, the development of scalable and cost-effective school-based interventions that successfully promote physical fitness among children and adolescents remains a large gap requiring international research focus over the next decade [69–71].

### Priority 5: Develop Universal Health-Related Fitness Cut-Points

The World Health Organization led major efforts to establish universal health-related cut-points for body mass index to detect overweight and obesity among children and adolescents aged 5–19 years [72]. For waist circumference, the age- and sex-specific 90th percentile has been proposed as an international cut-point to detect central obesity among children and adolescents aged 6–18 years [73]. Less international consensus exists for other measures of physical fitness. In 2016, Ruiz et al. conducted a meta-analysis of health-related cut-points for CRF and identified values of 42 and 35 mL/kg/min for boys and girls, respectively [74]. A major limitation of the Ruiz meta-analysis was a lack of age-specific cut-points. A more recent systematic review concluded that the variability in published CRF cut-points precludes the ability to identify universal age- and sex-specific cut-points [75]. There is a need for future studies using standardized CRF measures and similar health outcomes to improve the ability to identify universal sex- and age-specific CRF cut-points. There is a similar need for standardized measures of MSF to reduce heterogeneity in conducting meta-analyses for universal cut-points [76]. There is also a need for consensus on appropriate scaling methods to help account for body size when measuring physical fitness, which might be an important first step before developing universal health-related fitness cut-points.

### Priority 6: Investigate Interventions to Improve Fitness

Aside from school-based interventions, home-, family-, and community-based interventions could complement the promotion of physical fitness among children and adolescents [77, 78]. However, home-, family-, and community-based interventions have received less attention in the literature, with a particular gap existing for interventions targeting physical fitness as the primary outcome [79]. Most home-, family-, and community-based intervention studies have focused on physical activity levels as the primary outcome [79]. In addition, web-based or app-based interventions for health promotion have gained attention more recently [80, 81]. These types of studies are promising, especially as the world continues to grapple with the unique challenges that children and adolescents have faced because of the COVID-19 pandemic [82].

### Priority 7: Assess the Reliability and Validity of Fitness Measures

Reliability and validity are used to evaluate the quality of a fitness test and have important implications for fitness surveillance, the assessment of fitness-enhancing polices and interventions, and for linking fitness components to health outcomes. Existing tools and frameworks are available to help evaluate the quality of outcome measures [83]. Several comprehensive systematic reviews of the reliability [84, 85] and criterion validity of field-based fitness tests have been published [84, 86–88]. Reliability and validity data from these reviews have been used to develop field-based fitness test batteries for population health surveillance among children and adolescents. For example, information on the health-related predictive validity, criterion validity, reliability, and feasibility of field-based fitness tests was used to develop the ALPHA (Assessing Levels of Physical Activity) health-related fitness test battery for children and adolescents [84]. The ALPHA recommends the 20-m shuttle run test for CRF, handgrip strength and standing broad jump tests for MSF, and height, body mass, waist circumference, and skinfolds (triceps and subscapular) for body composition. Despite the widespread evidence regarding the reliability and validity of many fitness tests for school-aged children, few studies have validated fitness tests for preschoolers and school-aged children from low- and middle-income countries [89–91]. A better understanding of the criterion validity of field-based MSF tests (where appropriate laboratory-based criterion measures are used), and the reliability and validity of motor fitness tests (speed, agility, balance, coordination), is required [92].

### Priority 8: Develop a Common/Universal International Field-Based Fitness Test Battery

Fitness test batteries include a variety of standardized fitness measures often covering several components (e.g., CRF, MSF, body composition) that collectively indicate an individual’s overall physical fitness level. Worldwide, there are more than 15 field-based fitness test batteries for children and adolescents [93]. The most commonly used include the FitnessGram^®^ [94], Eurofit [95], and ALPHA [84] test batteries [31]. Therefore, it is challenging to pool data internationally given the difficulty of standardizing fitness test performances (e.g., because of differences in tests/protocols, performance metrics, age metrics, reporting procedures). There is a pressing need for collaboration to develop a universal field-based fitness test battery that can be implemented internationally. A scalable test battery requires a set of measures that are easily implemented with non-specialized personnel, have evidence of operating at a large scale, are effective (i.e., valid, reliable, high completion rate), and low cost [30]. A widely accepted protocol (e.g., core outcome set) for reporting results is also required, an issue that has been discussed in detail elsewhere [6, 96].

### Priority 9: Investigate and Reduce Inequalities in Fitness

Evidence from international comparison studies suggest that trends in CRF [35], standing broad jump [36], and sit-up performance [37] among children and adolescents have declined substantially since the start of the millennium. Some research suggests that the country trends in those with high fitness levels have not changed substantially, but trends in those with low fitness have declined substantially in more recent years, resulting in larger country-specific temporal inequalities among youth [97, 98]. There is also evidence that CRF varies substantially between countries, with the fittest children and youth residing in Africa and Northern Europe and those with the lowest fitness residing in South and Central America [99]. There is a need to address these inequalities both within (e.g., regional variations [54, 100]) and between countries to provide every child with the potential to attain healthy levels of physical fitness. An equity approach should always be implemented when investigating fitness, similar to approaches used in physical activity research [101]. However, scalable national and international approaches to reverse these fitness inequalities are unknown and represent a substantial area of future research.

### Priority 10: Develop an International Fitness Data Repository

There exist several international data repositories for physical activity, including the International Children’s Accelerometry Database (ICAD) [102], the Physical Activity Cohort Repository (PACE) [103], and the World Health Organization Global Health Observatory Data Repository for several health-related indicators, including body mass index and physical inactivity [104]. These data repositories provide easy access to aggregate data for harmonization by region or country, and they promote standardized data collection within countries for certain measures. The European FitBack project is an important effort that could evolve into a new international fitness data repository [105]. However, there remain issues with retaining data submitted through the FitBack portal, and with allowing researchers to access these raw data for research purposes. Future work is needed to expand existing platforms or to create a new data repository that can mirror efforts in physical activity and body mass index.

### Strengths and Limitations

This study has many strengths including a broad international representation of experts, the use of purposive and systematic sampling procedures to identify experts, a twin-panel design to cross-validate priorities, the use of a Delphi method with participant blinding, and three structured rounds of data collection. The findings from our study are the subjective opinion of the expert panel and may not represent the opinions of other experts who were not included in this study. During the panel 1 international meeting, content from the round 1 survey (i.e., the most reported priority areas identified by the panel) were discussed and may have introduced bias during round 2 responses. However, this bias was likely small given the strong agreement between panels. Most of the participants in panel 1 were Canadian, and we had limited representation from low- and middle-income countries and countries in Africa. Including more experts from these regions may have identified different priorities. It is also important to note that research is constantly evolving, and priorities may change in the future. For this reason, it will be important to revisit this Delphi exercise in the next decade to examine what work has been done and to update the international priorities in this area of research and surveillance.

## Conclusions

Using a systematic Delphi twin-panel approach with an international group of experts, we identified the top 10 international areas for physical fitness research and surveillance over the next decade. Priorities included, among others, the use of longitudinal studies, fitness surveillance to inform decision making, international fitness testing using valid, reliable and standardized measures, and the development of interventions to improve fitness among children and adolescents. The priorities identified in this study should help guide international collaborations and research efforts over the next decade and beyond.

## Supplementary Information

Below is the link to the electronic supplementary material.

Supplementary file1 (DOCX 43 KB)
