# Supplementary material for: Top 10 International Priorities for Physical Fitness Research and Surveillance Among Children and Adolescents: A Twin-Panel Delphi Study
Source: Sports Med. 2022 Aug 24;53(2):549–64. doi: 10.1007/s40279-022-01752-6 (PMC9399984; doi:10.1007/s40279-022-01752-6)
Supplement: Supplementary file 1 — Supplementary file1 (DOCX 43 KB) [file 40279_2022_1752_MOESM1_ESM.docx]

**Supplementary materials**

**Title**

Top 10 international priorities for physical fitness research and surveillance among children and adolescents: A twin-panel Delphi study

**Journal**

Sports Medicine

**Authors**

Justin J. LANG^1,2,3^, Kai ZHANG^3,4^, César AGOSTINIS-SOBRINHO^5^, Lars Bo ANDERSEN^6^, Laura BASTERFIELD^7^, Daniel BERGLIND^8^, Dylan O. BLAIN^9^, Cristina CADENAS-SANCHEZ^10^, Christine CAMERON^11^, Valerie CARSON^12^, Rachel C. COLLEY^13^, Tamás CSÁNYI^14,15^, Avery D. FAIGENBAUM^16^, Antonio GARCÍA-HERMOSO^17^, Thayse Natacha Q. F. GOMES^18^, Aidan GRIBBON^19^, Ian JANSSEN^20,21^, Gregor JURAK^22^, Mónika KAJ^23^, Tetsuhiro KIDOKORO^24^, Kirstin N. LANE^25^, Yang LIU^26,27^, Marie LÖF^28,29^, David R. LUBANS^30^, Costan G. MAGNUSSEN^31–34^, Taru MANYANGA^35^, Ryan McGRATH^36,37^, Jorge MOTA^38^, Tim OLDS^39,40^, Vincent O. ONYWERA^41^, Francisco B. ORTEGA^42,43^, Adewale L. OYEYEMI^44^, Stephanie A. PRINCE^1,2^, Robinson RAMÍREZ-VÉLEZ^17,45,46^, Karen C. ROBERTS^1^, Lukáš RUBÍN^47,48^, Jennifer SERVAIS^19^, Diego Augusto Santos SILVA^49^, Danilo R. SILVA^18^, Jordan J. SMITH^30^, Yi SONG^50^, Gareth STRATTON^51^, Brian W. TIMMONS^3,52^, Grant R. TOMKINSON^39,53^, Mark S. TREMBLAY^3,54,55^, Stephen H. WONG^56^, Brooklyn J. FRASER^34^

**Affiliations**

1. Centre for Surveillance and Applied Research, Public Health Agency of Canada, Ottawa, ON, Canada
2. School of Epidemiology and Public Health, University of Ottawa, Ottawa, ON, Canada
3. Healthy Active Living and Obesity Research Group, Children’s Hospital of Eastern Ontario Research Institute, Ottawa, ON, Canada
4. School of Human Kinetics, University of Ottawa, Ottawa, ON, Canada
5. Faculty of Health Sciences, Klaipeda University, Klaipeda, Lithuania
6. Department of Sport, Food and Natural Sciences. Western Norway University of Applied Science.
7. Population Health Sciences Institute, Newcastle University, Newcastle upon Tyne, UK
8. Department of Global Public Health and Centre for Epidemiology and Community Medicine (CES), Karolinska Institutet, Stockholm, Sweden
9. Institute of Management and Health, University of Wales Trinity Saint David, Wales, United Kingdom
10. PROFITH “PROmoting FITness and Health through physical activity” Research Group, Sport and Health University Research Institute (iMUDS), Department of Physical and Sports Education, Faculty of Sport Sciences, University of Granada, Granada, Spain.
11. Canadian Fitness and Lifestyle Research Institute, Ottawa, ON, Canada
12. Faculty of Kinesiology, Sport, and Recreation, University of Alberta, Edmonton, AB, Canada
13. Health Analysis Division, Statistics Canada, Ottawa, ON, Canada
14. Faculty of Primary and Pre-School Education, ELTE Eötvös Loránd University, Budapest, Hungary
15. Institute of Teacher Education, Hungarian University of Sports Science, Budapest, Hungary
16. Department of Health and Exercise Science, The College of New Jersey, Ewing, NJ, USA
17. Navarrabiomed, Hospital Universitario de Navarra (HUN), Navarra Institute for Health Research (IdiSNA), Universidad Pública de Navarra (UPNA), Pamplona, Navarra, Spain.
18. Department of Physical Education, Federal University of Sergipe, São Cristóvão-SE, Brazil
19. Centre for Population Health Data, Statistics Canada, Ottawa, ON, Canada
20. School of Kinesiology and Health Studies, Queen’s University, Kingston, ON, Canada
21. Department of Public Health Sciences, Queen’s University, Kingston, ON, Canada
22. Faculty of Sport, University of Ljubljana, Ljubljana, Slovenia
23. Hungarian School Sport Federation, Hungary
24. Research Institute for Health and Sport Science, Nippon Sport Science University, Tokyo, Japan
25. School of Exercise Science, Physical and Health Education, University of Victoria, Victoria, BC, Canada
26. School of Physical Education and Sport Training, Shanghai University of Sport, Shanghai, China
27. Shanghai Research Center for Physical Fitness and Health of Children and Adolescents, Shanghai University of Sport, Shanghai, China
28. Department of Biosciences and Nutrition, Karolinska Institutet, Sweden
29. Department of Health, Medicine and Caring Sciences, Linköping University, Sweden
30. Centre for Active Living and Learning, College of Human and Social Future, University of Newcastle, Newcastle, Australia
31. Baker Heart and Diabetes Institute, Melbourne, Victoria, Australia
32. Research Centre of Applied and Preventive Cardiovascular Medicine, University of Turku, Turku, Finland.
33. Centre for Population Health Research, University of Turku and Turku University Hospital, Turku, Finland.
34. Menzies Institute for Medical Research, University of Tasmania, Hobart, Tasmania, Australia.
35. Division of Medical Sciences, University of Northern British Columbia, Prince George, BC, Canada
36. Department of Health, Nutrition, and Exercise Sciences, North Dakota State University, Fargo, ND, US.
37. Fargo VA Healthcare System, Fargo, ND, USA
38. Research Center in Physical Activity, health and Leisure (CIAFEL)-Faculty of Sports-University of Porto (FADEUP) and Laboratory for Integrative and Translational Research in Population Health (ITR), Porto, Portugal
39. Alliance for Research in Exercise, Nutrition and Activity (ARENA), University of South Australia, Adelaide, SA, Australia
40. Murdoch Children’s Research Institute, University of Melbourne, Parkville, Melbourne, VIC, Australia
41. Department of Physical Education, Exercise and Sports Science, Kenyatta University, Nairobi, Kenya
42. PROFITH “PROmoting FITness and Health Through Physical Activity” Research Group, Sport and Health University Research Institute (iMUDS), Department of Physical and Sports Education, Faculty of Sport Sciences, University of Granada, 18071 Granada, Spain.
43. Faculty of Sport and Health Sciences, University of Jyväskylä, Jyväskylä, Finland.
44. Department of Physiotherapy, University of Maiduguri, Maiduguri, Nigeria
45. Navarrabiomed, Hospital Universitario de Navarra (HUN)-Universidad Pública de Navarra (UPNA), IdiSNA, Pamplona, Spain
46. CIBER of Frailty and Healthy Aging (CIBERFES), Instituto de Salud Carlos III, Madrid, Spain
47. Department of Physical Education and Sport, Technical University of Liberec, Liberec, Czech Republic
48. Institute of Active Lifestyle, Palacký University Olomouc, Olomouc, Czech Republic
49. Sports Center, Federal University of Santa Catarina, Florianópolis, SC, Brazil
50. Institute of Child and Adolescent Health, School of Public Health, Peking University; National Health Commission Key Laboratory of Reproductive Health, Beijing, 100191, China
51. Applied Sport Technology Exercise and medicine Research Centre, Faculty Science and Engineering. Swansea University. Wales. UK
52. Child Health & Exercise Medicine Program, McMaster University, Hamilton, ON, Canada
53. Department of Education, Health and Behavior Studies, University of North Dakota, Grand Forks, ND, USA
54. Department of Pediatrics, University of Ottawa, Ottawa, ON, Canada
55. Department of Health Sciences, Carleton University, Ottawa, ON, Canada
56. Department of Sports Science & P.E., The Chinese University of Hong Kong

File 1 – Meeting details, August 19th, 2021

Meeting schedule

| **Estimated time (EDT)** | **Activity** |
| --- | --- |
| 7:00 – 7:15 | Justin Lang – Opening remarks |
| 7:15 – 7:30 | Brooklyn Fraser – Presentation 1  “Health-related criterion-referenced cut-points for physical fitness among youth: two systematic reviews” |
| 7:30 – 7:35 | Questions and answers for Brooklyn |
| 7:35 – 7:50 | Nick Seguin – Presentation 2  “Health-related criterion-referenced cut-points for the 20-meter shuttle run test among children and youth – an exploration and meta-analysis” |
| 7:50 – 7:55 | Questions and answers for Nick |
| 7:55 – 8:10 | Tamás Csányi – Presentation 3  “The effect of Daily PE on adolescents’ health-related physical fitness – national preliminary results from Hungary” |
| 8:10 – 8:15 | Questions and answers for Tamás |
| 8:15 – 8:30 | Gregor Jurak – Presentation 4  “FitBack – The European Network for the Support of Development of Systems for Monitoring Physical Fitness of Children and Adolescents” |
| 8:30 – 8:35 | Questions and answers for Gregor |
| 8:35 – 8:45 | Break and transition |
| 8:45 – 10:45 | Overview of the Delphi study  Preliminary results and discussion |
| 10:45 – 11:00 | Justin Lang – Closing remarks |

List of invitees

- **Statistics Canada**
  - Jennifer Servais, Aidan Gribbon, and the CHMS team
- **Public Health Agency of Canada**
  - Surveillance, Policy and Program Divisions
- **Canadian Fitness and Lifestyle Research Institute**
- **Canadian Fitness Experts**
  - Mark Tremblay, CHEO RI
  - Rachel Colley, Statistics Canada
  - Ian Janssen, Queen’s University
  - Brian Timmons, McMaster University
  - Luc Léger, Université de Montréal
  - Valerie Carson, University of Alberta
  - Jonathan McGavock, University of Manitoba
  - Jennifer Reed, University of Ottawa Heart Institute
  - Taru Manyanga, University of Northern British Columbia
  - Kristin Lane, University of Victoria
  - Kai Zhang, University of Ottawa (PhD student)
  - Nick Seguin, Carleton University (MSc student)
- **International Fitness Experts**
  - Brooklyn Fraser, Menzies Institute for Medical Research (Australia)
  - Grant Tomkinson, University of North Dakota (USA)
  - Diego Silva, Federal University of Santa Catrina (Brazil)
  - Cristina Cadenas-Sanchez, University of Granada (Spain)
  - Jordan Smith, University of Newcastle (Australia)
  - Laura Basterfield, Newcastle University (United Kingdom)
  - Stephen Wong, Chinese University of Hong Kong (Hong Kong)
  - Yang Liu, Shanghai University of Sport (China)
  - Tetsu Kidokoro, Nippon Sport Science University (Japan)
  - Vincent Onywera, Kenyatta University (Kenya)
  - Adewale Oyeyemi, University of Maiduguri (Nigeria)
  - Tamas Csanyi, Hungarian School Sport Federation (Hungary)
  - Monika Kaj, University of Pécs (Hungary)
  - Gregor Jurak, University of Ljubljana (Slovenia)
  - Gavin Sandercock, University of Essex (United Kingdom)
  - Gareth Stratton, Swansea University (United Kingdom)

File 2 - Responses provided during round 1

Question: In your opinion, what is the most important international priority area for physical fitness research and surveillance among children and youth that should be addressed over the next 10 years? Please describe the priority area in one or two sentences.

Notes: Italicized priority areas were common between both panels.

**Panel 1**

1. *Conduct longitudinal studies to assess changes in fitness and associations with health*
   1. “Longitudinal study examining the changes on physical fitness of children and youth especially focusing on the cardiovascular fitness.”
   2. “Prospective studies of the independent effects of CRF/MSF on health outcomes from childhood into adulthood.”
   3. “Prospective, longitudinal research looking at relationship of PA, fitness, etc. on health outcomes from child to middle aged adult.”
2. *Use fitness surveillance to inform decision making*
   1. “Communicating the importance of fitness as a product measure that is an indicator of sound growth and development as well as of health to policy and strategy makers.”
   2. “Determining how fitness research and surveillance efforts can most effectively dovetail with the needs/goals of end users.”
   3. “To invest in research partnerships and infrastructure that values share and use fitness surveillance data to make immediate impact.”
3. Investigate interventions to improve fitness
   1. “Developing personalized PA interventions based on PF and 24-HMB and supported by AI.”
   2. “Development of effective/workable population-based strategies/interventions to increase the CRF/MSF of children/youth to improve their long-term health.”
   3. “Evaluation of intervention for promoting physical fitness among children and youth. To study the effects of interventions for promoting physical fitness among children and youth.”
   4. “How and which kind of school-based PF interventions are effective in middle and long- term perspective. What are the core educational tools and methods for interiorizing the relevant skills, knowledge and attitudes for the current and future health behaviors?”
   5. “Investigate what interventions are most effective at affecting a change in fitness levels
   6. “Targeted physical fitness interventions, and public health promotion.”
   7. “Training efficiency for all lifestyle habits. i) Adequate training principles for child and adolescents to improve all aspect of fitness; ii) Intervention programs at school and all governments levels.”
4. Assess the reliability and validity of fitness measures
   1. “Development of a reliable and valid test of aerobic fitness for younger children and youth.”
   2. “Testing. Adequate metrological aspects of testing (protocol, validity, reliability, accessibility, sensibility, specificity…) are essential to demonstrate the links between health risks and morbidity and to assess the relative and respective importance between fitness ((aerobic, strength, power…) motor abilities, body composition, and physical activity and nutrition profiles.”
   3. “Valid measures.”
5. Develop a common/universal international field-based fitness test battery
   1. “Assessment battery of physical fitness among children and youth. To develop a simple, reliable and valid assessment battery of physical fitness of children and youth, which can be accepted and used in most countries.”
   2. “Consistency in measures.”
   3. “Determining an international consensus on the most appropriate fitness measures to be included in national health surveillance, and cooperating to conduct a coordinated effort to regular monitor and report upon the fitness of children and adolescence.”
   4. “Develop a number of consistent measures.”
   5. “Development of a global fitness test battery for use among 3–8-year-olds in all countries.”
   6. “Development of a global fitness test battery for use among 9–17-year-olds in all countries.”
   7. “Establishing a global consensus protocol/battery of fitness tests for children & youth that can be adopted worldwide and are accessible.”
   8. “Establishment of accepted approaches to measuring fitness that can be applied in a wide range of countries - high, middle and low income.”
   9. “Establishment of country specific and culturally sensitize, objective and robust fitness testing batteries.”
   10. “Harmonization of methodology for existing tests.”
   11. “Identification of standardized protocols to measure physical fitness.”
   12. “International consensus on core set of measures that require little equipment.”
   13. “Reliable and internationally comparable and feasible measures of physical fitness.”
   14. “The development of a consensus statement (endorsed by relevant organizations, perhaps including WHO) for which field-based CRF test(s) and indicator(s) to use in international surveillance. Indicators may include a move away from predicted values to raw values.”
   15. “Encourage the performance of field tests for cardiorespiratory fitness.”
6. Investigate and reduce inequalities in fitness
   1. “Reduce inequality in fitness.”
   2. “Structural Inequities in activity/fitness.”
   3. “Understanding the main factors that explain low activity levels among non-white girls.”
7. *Implement regular and consistent international/national fitness surveys using common measures*
   1. “An international health/fitness survey of children/youth inclusive of low-, middle- and high-income countries from all world regions (using standardized test procedures similar to the HELENA study in Europe).”
   2. “Developing and executing a global fitness survey for children 3-8 years of age - boys and girls, low-, middle, and high-income countries, with urban and rural samples across family SES.”
   3. “Developing and executing a global fitness survey for children 9-17 years of age - boys and girls, low-, middle, and high-income countries, with urban and rural samples across family SES.”
   4. “Regular, consistent and systematic collection of nationally representative physical fitness data.”
8. Develop an international fitness data repository
   1. “A need for an international repository of standardized fitness test results to improve comparability between nations and over time.”
   2. “Developing an open access database of international data.”
   3. “Data sharing system (making the raw data of fitness testing publicly available).”
   4. “Establishing international database of children, adolescent and adults’ physical fitness.”
9. *Develop universal health-related fitness cut-points*
   1. “Criterion standards.”
   2. “Definition of cutoff points for cardiorespiratory fitness.”
   3. “Determining what levels of health-related fitness (CRF, MF) are linked to health outcomes in youth.”
   4. “Development of universal criterion-referenced cut-points in cardiorespiratory fitness (CRF) and musculoskeletal (MSF) associated with health outcomes among children/youth (e.g., as part of an international health/fitness survey) that consider age, gender, growth, and maturation.”
   5. “Development of universal health-based fitness standards for ages and sexes and different areas of fitness (e.g., cardiorespiratory fitness, muscular strength).”
   6. “Fitness guidelines.”
   7. “Physical fitness guidelines.”
   8. “The development of guidelines for cardiorespiratory fitness among children and youth similar to the 24-Hour Movement Guidelines for use in population health surveillance (e.g., % meeting CRF recommendations) and evaluation of public health interventions. Additionally, it would be beneficial to have recommendations for specific clinical populations (e.g., obesity, T1 diabetes, MetS).”
   9. “There is a need to develop international criterion reference standards for low cost and easy to administer field-based fitness tests (e.g., 20 m shuttle run test) for use in surveillance.”
10. Increase fitness data in low- and middle-income countries
    1. “Establishment of physical fitness data base for Kenyan children and youth aged 3 to 17 years.”
    2. “Continuous monitoring of physical fitness among Kenyan children and youth aged 3 to 17 years.”
    3. “Improving surveillance of attributes of physical fitness among children and youth in the LMICs.”
11. *Better understand how components of fitness impact health*
    1. “Better understanding of how different components of muscle strength and endurance impact health.”
12. Identify the dose-response relationship between fitness and health
    1. “Dose-response relationships between CRF/MSF and physical activity and health outcomes.”
13. Use fitness as a clinical vital sign to monitor for health screening in clinical settings
    1. “Disseminate the theme of cardiorespiratory fitness for health services.”
    2. “Fitness as a medical record to follow-up from preschool ages to elderly.”
    3. “Fitness as a tool for health screening.”
14. *Develop international/national normative-referenced centile values for fitness*
    1. “Creating international and regional physical fitness normative values.”
    2. “Creating/establishing norms/predictive equations for the consensus gold standard tests in children and youth.”
    3. “Fitness norms for children, especially younger children.”
    4. “Establishment of international comparisons of fitness among children and youth.”
    5. “International comparison of physical fitness status among children and youth. To reveal the status of physical fitness among children and youth and provide general results of a certain region/country among regions/countries.”
15. Untangle the health benefits of fitness vs. physical activity
    1. “Teasing out the benefits of fitness from physical activity and teasing out the differential benefits of muscular fitness and aerobic fitness in terms of outcomes such a psychological well-being and academic attainment. The magnitude of these benefits is difficult to ascertain without large-scale surveillance and research appropriately controlling for baseline variability and confounders such a social economic status.”
    2. “Untangling the health benefits of fitness vs. physical activity.”
16. *Implement school-based fitness monitoring*
    1. “Engagement with schools to collect standardized data.”
    2. “National or state level, yearly, standardized health-related physical fitness monitoring in schools as an integrated part of the fitness education process and school health services. Updating and cross validating different fitness standards and its correlation with different health criteria.”
17. Use fitness as a primary outcome in research studies that intervene with physical activity
    1. “To move away from measures of ‘physical activity’ as the primary outcome in surveillance and interventions (reduce or eliminate the use of self-report and proxy-reported activity entirely). Move toward treating physical activity as the (input dependent variable) and objective measures of physical and psychological health as the primary outcomes (independent variables).”
18. Shift from a focus on obesity to a focus on fitness for health
    1. “We need to turn the spotlight away from obesity by further illustrating that the benefits of fitness from physical activity are independent of body mass index and hopefully show that fitness is a stronger, more potent predictor of health and well-being than fatness.”
19. Study the link between fitness and mental/cognitive health
    1. “Development of research on the relationship between cardiorespiratory fitness and mental health in all age groups.”
    2. “Fitness research: What are the causal links between different components of physical fitness (e.g., cardio-respiratory, musculoskeletal) and mental health (both well-being and ill-being) independently of each other (and other confounders).”
    3. “Physical fitness components and its role in different cognitive functions.”
    4. “Psychological determinants of healthy and risky physical fitness state of youth.”
20. Identify determinants and correlates to help improve fitness among children and youth
    1. “The determinants and/or correlates of physical fitness among children and youth. To study on what expose factors can influence physical fitness among children and youth and to what extent they can influence physical fitness.”
21. Measure fitness to help understand physical activity levels in a population
    1. “Explain to the scientific community that measuring cardiorespiratory fitness is a way to directly measure people's physical activity level.”
22. Overcome the stigma of fitness testing (i.e., fear of injury)
    1. “Overcoming one of the educational and philosophical beliefs that fitness assessment has a negative impact on participants, is a bad experience particularly in the low fit children’s groups.”
23. Implement an international fitness survey for those with disabilities
    1. “Adaptation of research priorities 1-4 for children with various disabilities/limitations.”
24. Identify the main construct measures of fitness among children and youth
    1. “What are the main indicators of physical fitness among children and youth.”
25. Improve international comparison of fitness trends
    1. “International comparison of physical fitness trend among children and youth. To compare physical fitness trend among children and youth all over the world.”
26. Investigate the associations between motor fitness and health
    1. “Explore causality between motor fitness and health outcomes in adulthood.”
27. Investigate fitness as a mediator of obesity risk
    1. “Role of physical fitness as mediator of the obesity risk reduction.”
28. Assess trends in fitness while controlling for adiposity
    1. “Analyzing effect of secular trends in fitness when controlling for anthropometric measures.”
29. Determine the frequency that fitness should be measured in a population
    1. “Determine the frequency that fitness should be measured in this population.”
30. Assess the effect of COVID-19 restrictions on fitness levels
    1. “Identifying effect of COVID 19 restrictions on children fitness to address this problem to policy makers.”
31. Promote the benefits of resistance type training
    1. “Promoting equivalents for the benefits of resistance type training in children with the current bias toward evaluation only of aerobic type activities as inputs and the accompanying focus on aerobic fitness as an outcome.”
32. Investigate international trends in obesity
    1. “Global trend in overweight and obesity and its association with the different diseases.”
33. Develop field tests that are independent of body size
    1. “To develop a field test that is independent of body size and applicable across the ages of 3 to 17 years.”
34. Investigate the role of genetics and the environment on fitness
    1. “Study the individual differences, role of the genetics and environmental constraints of the PF state and the effect of these determinants on current and future health status.”
35. Identify backup fitness measures to use as a proxy when primary measures can’t be used
    1. “Should one of the consensus tests not be able to be administered, establishing a good proxy test (or secondary test) that could be used instead.”
36. Use fitness testing for sport talent identification
    1. “Use of fitness tests for sporting talent identification and development.”

**Panel 2**

1. *Conduct longitudinal studies to assess changes in fitness and associations with health*
2. “The importance of long-term high fitness in relation to development of obesity and other metabolic disorders.”
3. “Surveillance of specific components of physical fitness throughout childhood and adolescence.”
4. “We need to elaborate on the dose response relationship and prospective associations between fitness and health in young adulthood in a broad perspective.”
5. “More studies on physical fitness in early childhood and later health outcomes.”
6. “Maybe is to emphasize the relationship between physical fitness and all health indicators since preschool. Children need to be involved in physical activity since the first year of life, and again my opinion school is the right place to involve children in several activities. So, we need to improve research in these areas, to understand which kind of activities need to be included in PE classes to improve motor development and physical fitness.”
7. *Implement regular and consistent international/national fitness surveys using common measures*
8. “Internationally consistent population-level surveillance.”
9. “Establish harmonized monitoring systems for PA and fitness to increase comparability between countries and regions.”
10. “For surveillance we need to standardize a good field test. At the moment most studies use their own and comparison between studies is difficult.”
11. “The first priority one is to set standard indicators and measurements for physical fitness, although many researchers focus on physical fitness, such as cardiovascular endurance, muscular strength and endurance, flexibility, and body composition, the uniform indicators are still needed.”
12. “To develop standard and comparable tests around the world.”
13. “National (Australian) and international monitoring of young people's MSF.”
14. “National (Australian) and international monitoring of young people's CRF.”
15. “The second priority one is to set up regular national surveillance of physical fitness for children and adolescents. Although several countries, such as Japan, Korea, China, France etc., to some extent have national surveillance, little of them form a system to insist for long time.”
16. “To be able to regularly monitor children's (preschoolers up to adolescents) physical fitness to assess trends over time.”
17. *Develop universal health-related fitness cut-points*
18. “Evidence-based cut-offs for fitness levels in children that impact meaningful outcomes, both short and long-term.”
19. “Cut-off points to determine low levels of physical fitness, mainly of muscular strength.”
20. *Study the link between fitness and mental/cognitive health*
21. “Relationship between physical fitness parameters and mental health.”
22. “Explore the dose-dependent relationship between physical fitness and mental health outcomes in youth (e.g., anxiety, depression), and identify the most effective interventions for treating mental ill-health in youth.”
23. “Physical fitness and neurodevelopment.”
24. *Develop international/national normative-referenced centile values for fitness*
25. “To developed reference values/measures for physical fitness components surveillance across countries (i.e., low-middle-high income) across life span.*”*
26. Implement scalable school-based interventions to improve and promote fitness
27. “Scalable school-based interventions to improve young people's musculoskeletal fitness (MSF).”
28. “In my opinion, the most important international priority area for physical fitness research is to show over the next year the fundamental role of physical education classes in the promotion of physical fitness. In my country, most children and adolescents have access to physical activity only in school.”
29. “Scalable school-based interventions to improve young people's cardiorespiratory fitness (CRF).”
30. “Strategies to best improve physical fitness early in life with targeted interventions via skills, knowledge, services, etc. that address the needs of modern-day youth.”
31. “We need to elaborate on scaling up interventions target to increase physical fitness in the young populations.”
32. “Health education on physical fitness.”
33. “The fifth one is to combine physical fitness research into health lifestyle, and extent its implication in schools.”
34. *Use fitness surveillance to inform decision making*
35. “What policy level actions are needed to improve physical fitness in youth, and what is the return on investment? What happens if current trends continue? Who pays?”
36. Focus on shifting trends in fitness levels among children
37. “Shifting trends in fitness and physical activity/sedentary behaviors in children, particularly in a peri and post (or "advanced peri" perhaps) COVID-19 pandemic world.”
38. Investigate cost effectiveness of interventions aimed at increasing fitness
39. “Cost effectiveness analyses of certain behavioral interventions to increase fitness/PA both among the general population and 'at-risk' populations.”
40. “Effect of fitness training on body systems other than the cardiovascular, musculoskeletal, and neuromuscular systems (e.g., brain, liver/kidney).”
41. “Speed-agility parameter.”
42. “We need RCT studies to elucidate health improvements related to increase in different types of fitness.”
43. “Sustainable, population-level programs to increase aerobic and muscular fitness in kids.”
44. Investigate the causal associations between fitness for health and well-being
45. “Causal role of fitness for health and well-being.”
46. Improve muscular strength promotion among youth
47. “Muscular strength promotion from early years.”
48. *Implement school-based fitness monitoring*
49. “Improve school system to integrate country-level surveillance systems around the world.”
50. Investigate effective interventions to improve fitness among unfit youth
51. “Identification of unfit children early in life with practical fitness tests and assessment of interventions that are most effective to increase fitness in unfit youth and maintain fitness in fit youth.”
52. Increase fitness data in low- and middle-income countries and rural areas
53. “Physical fitness in children from low-income countries and/or rural areas.”
54. Implement physical literacy interventions in schools with a focus on fitness
55. “I love the theoretical conception of Physical Literacy. Probably to improve intervention research included this conception and the role of this in physical fitness could be an interesting way to improve the central role of PE classes in school, and to improve physical fitness.”
56. *Better understand how components of fitness impact health*
57. “Muscular fitness.”
58. “The relationship with health indicators.”
59. “We need to include fitness in the concept of metabolic syndrome. Obesity has a central role mainly because it was a part of the definition of metabolic disorder such as metabolic syndrome. However, low fitness may even be worse than obesity in relation to unhealthy metabolism.”
60. “We should know more about muscular fitness in relation to health (metabolic and bone).”
61. “The fourth one is to continue explore the association between physical fitness and its health outcomes, especially for adolescents.”
62. Engage stakeholders, funding bodies, NGOs, etc. to understand the importance of fitness
63. “We need to better engage stakeholders, funding bodies, NGOs, etc. on the importance of this area.”
64. Tracking of fitness from childhood to late adolescence
65. “Tracking of physical fitness from childhood to late adolescence.”
66. Assess physical fitness by socioeconomic status and parental education
67. “Evaluation the physical fitness using stratification for socio economic status and parents’ education; and verify if a decline is real against the mass media mindset.”
68. Establishing consensus on how best to account for body size/shape when measuring fitness
69. “How fitness measures in youth are accounted for body size/shape.”
70. Determine if body composition or physical fitness is a better predictor of health outcomes
71. “Determining whether body composition or physical fitness is a better predictor of health in the pediatric population.”
72. Investigate the parental influence on childhood fitness levels
73. “To involve parents in our future intervention. In my opinion, we need to improve our future physical fitness intervention included parents, and improve parents’ involvement in sports aim to improve their physical fitness.”
74. Investigate the genetic determinants of physical fitness
75. “Genetics of fitness, including epigenetics.”
76. Investigate the relationship between sport participation and physical fitness
77. “Again, the place is school, and maybe to create several research areas about the relationship between sports and physical fitness and to improve public policies to offer several sports to engage children and adolescents in school after classes. The school need to be a place to evolve children and adolescents in several sports.”
78. Investigate the link between fitness and nutrition
79. “Nutrition and physical fitness.”
